# A Rare Cause of Exudative Pleural Effusion in a Female

**DOI:** 10.7759/cureus.16237

**Published:** 2021-07-07

**Authors:** Sherif T Abuserewa, Shawn Esperti, Thaddeus Golden, Richard Duff

**Affiliations:** 1 Department of Internal Medicine, Grand Strand Regional Medical Center, Myrtle Beach, USA; 2 Department of Pulmonary and Critical Care Medicine, Grand Strand Regional Medical Center, Myrtle Beach, USA

**Keywords:** yellow nail syndrome, exudative pleural effusion, dystrophic nails, rare lung disease, micro-vasculopathy, lymphatic drainage dysfunction

## Abstract

Yellow nail syndrome is an extremely rare syndrome that presents with a clinical triad of thickened yellow nails, lymphedema, and recurring pulmonary manifestations (pleural effusion, chronic cough, or bronchiectasis), usually in the population above the age of 50 years. We describe a case of yellow nail syndrome in a 48-year-old lady who presented with the typical classical triad of this syndrome.

## Introduction

Yellow nail syndrome is a very rare condition; less than 400 cases were reported since it was first described in 1964. Although there is a classic clinical triad of yellow dystrophic nails, lymphedema, and respiratory symptoms, only around 30% of cases present with the classical triad simultaneously (as in this case), while other cases usually present with individual symptoms that occur sequentially, and that’s why it is difficult to be diagnosed and needs a physician to be aware and mindful of the syndrome [1-3]. Its etiology is still unknown; however, involvement of the lymphatic system with possible lymphatic drainage dysfunction is suggested. It is usually managed symptomatically, while in some cases, it resolves spontaneously.

This case was presented as an e-poster at the American Thoracic Society (ATS) international conference in May 2021.

## Case presentation

A 48-year-old female presented to the emergency room (ER) with right-sided chest pain. She reported that over the past two months, she had intermittent fever up to 102 °F. She did see her primary care physician (PCP) in June who thought she had pneumonia and started her on levofloxacin. However, the patient had not experienced any cough, sputum production, wheezing, shortness of breath, chest pain, nausea, vomiting, diarrhea, constipation, chills, night sweats, or weight loss. Two days before admission, she started to have sharp, right-sided chest/back pain that would occur with deep breaths. She also endorsed swelling in her lower extremities. 

**Figure 1:**
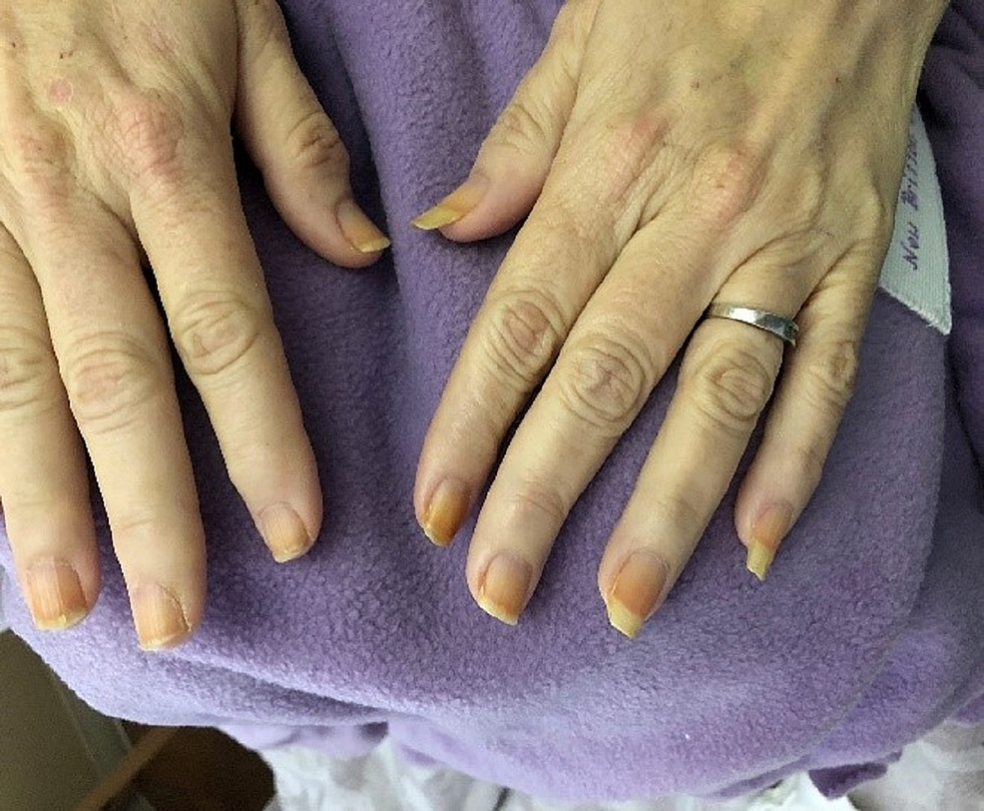
Yellow, thickened, and pitted fingernails

**Figure 2:**
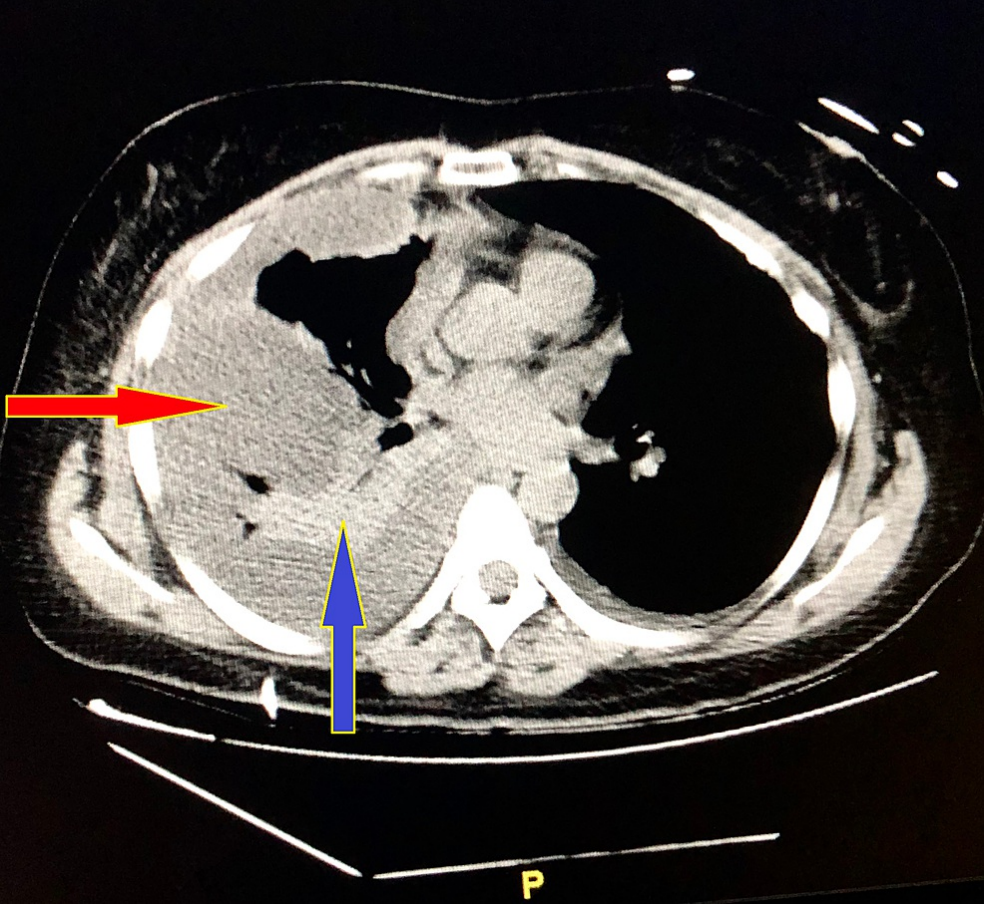
CT chest without contrast showing large right-sided encysted pleural effusion (red arrow) with collapsed lung (blue arrow)

On examination, she was alert and oriented to time, person, and self, febrile at 100.8 °F, tachycardic at 100s beats/min, tachypneic at 20s breaths/min, hypoxic, and on Venti mask 15 L with O2 saturation at 98%. Chest auscultation revealed bilateral symmetric expansion, left-sided breath sounds of good intensity, while right-sided breath sounds were diminished and associated with egophony. The patient’s nails were pitted, thickened, and yellowish in color (Figure 1). Computed tomography (CT) chest demonstrated loculated right pleural effusion and right lung consolidation/collapse (Figure 2).

**Figure 3:**
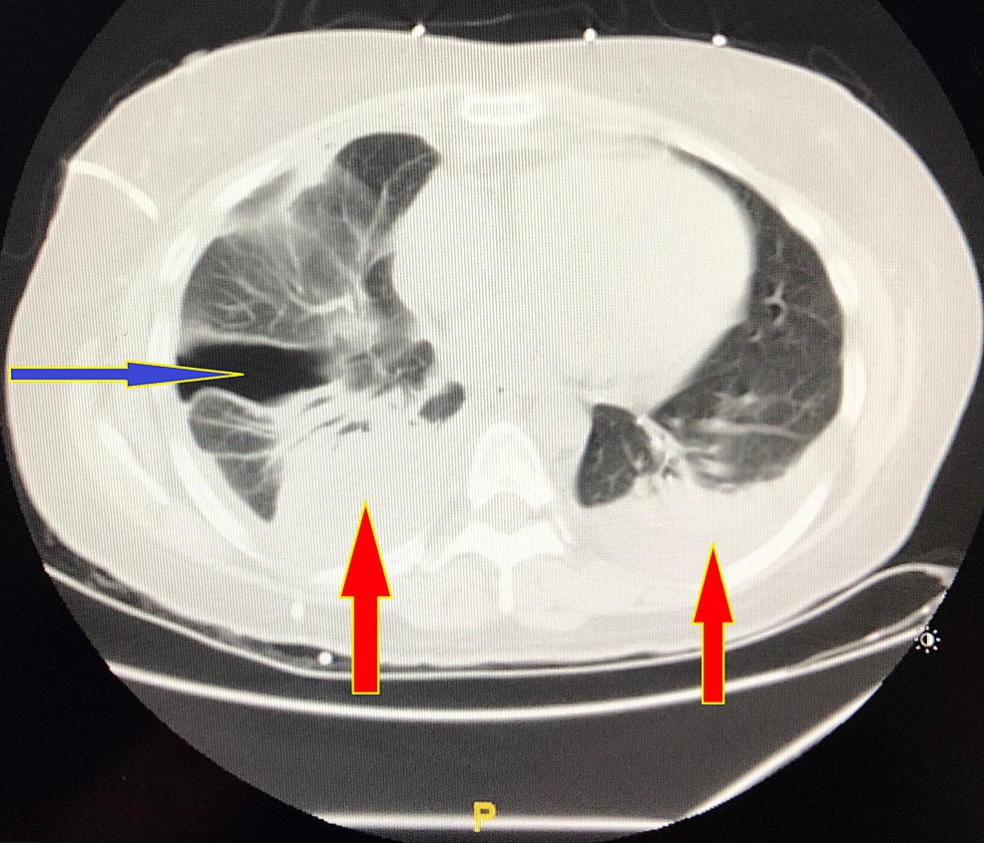
CT chest without contrast showing right-sided small encysted pleural effusion (red arrow on the right) with pneumothorax (blue arrow), and small, free, left-sided pleural effusion (red arrow on the left).

The patient was started on vancomycin and piperacillin-tazobactam, pulmonary toilet, and flutter valve. Thoracentesis was performed with pleural fluid analysis demonstrating a clear, yellowish exudate, 1026 white blood cells/mm3, 418 red blood cells/mm3, total protein 3.9 gm/dl, lactate dehydrogenase (LDH) 1117 units/L. Acid-fast bacilli, fungus smear and culture, anaerobic culture, Gram stain, and conventional cultures were all negative. Two pigtails were inserted and connected to -20 cm water suction and six doses of tpa/dornase injections were given for liquefaction of septations in the pleural space. Follow-up CT Chest showed a small right hydro-pneumothorax and significantly decreased amount of right pleural fluid (Figure 3).

Another set of all previous cultures and stains were repeated, which all again returned to be negative. Rheumatological work-up was done and the following markers were ordered: antinuclear antibody (ANA), rheumatoid factor (RF), cyclic citrullinated peptide (CCP), complement component 3 and 4 (C3, C4), scleroderma (Scl-70) antibody, anti-double-stranded DNA antibody (anti ds-DNA Ab), anti-smooth muscle antibody, anticardiolipin IgG, IgA, and IgM antibodies, lupus anticoagulant, erythrocyte sedimentation rate (ESR), and c-reactive protein (CRP). Furthermore, hepatitis panel including hepatitis A virus (HAV), hepatitis B virus (HBV), hepatitis C virus (HCV), and human immunodeficiency virus (HIV) were also ordered. ESR was >140, however, all rheumatological and hepatitis markers were negative.

**Figure 4:**
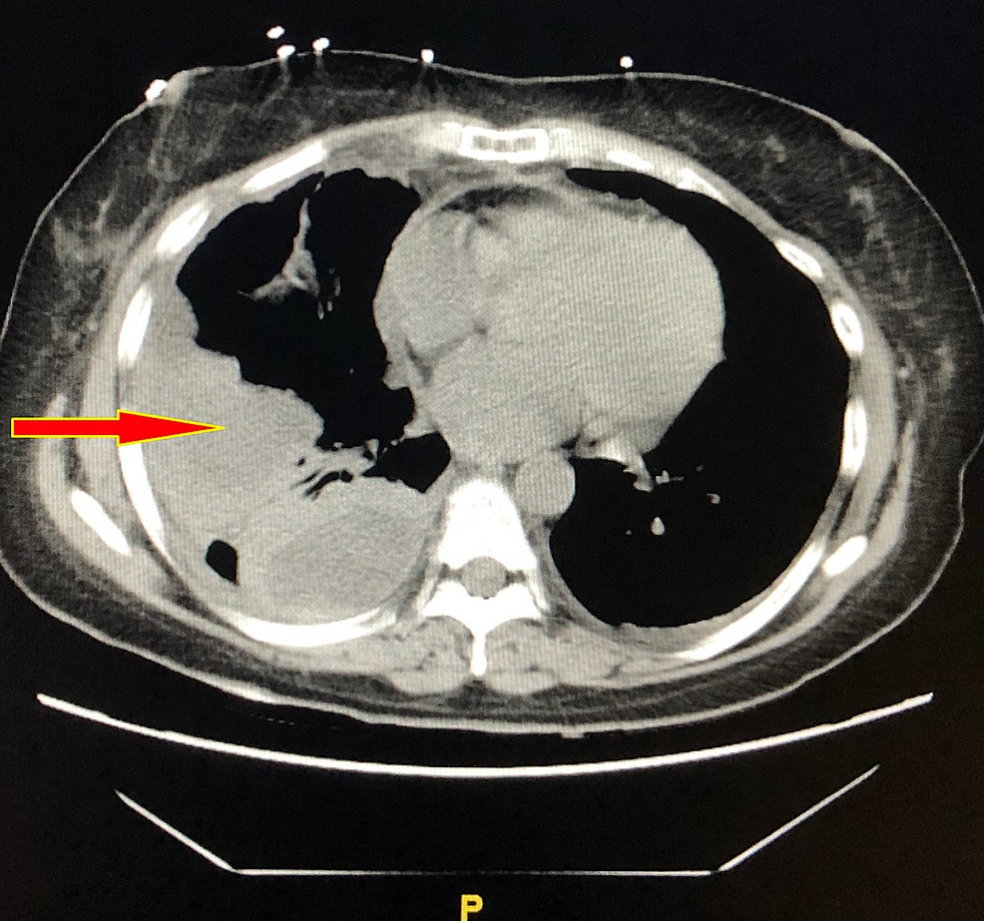
CT chest without contrast showing reaccumulating right-sided pleural effusion in comparison to Figure 3

After two consecutive days of low output from both pigtails, they were removed. However, the follow up computed tomography (CT) of the chest one day later showed rapid re-accumulation of the pleural fluid (Figure 4).

The patient then underwent video-assisted thoracoscopic surgery (VATS) and pleural biopsy followed by pleurodesis. Pleural biopsy was negative for malignancy and was only significant for inflammation and granulation tissue. Pleural fluid cytology was also negative for malignancy. Infectious disease consult was done who recommended narrowing the spectrum to ampicillin-sulbactam only in the presence of clinical improvement and negative pleural fluid and blood cultures. Later on, patient was started on high doses of vitamin E with marked improvement in her symptoms.

## Discussion

Yellow nail syndrome is a rare clinical triad of yellow nail discoloration and dystrophy, lymphedema, and recurrent pulmonary symptoms. Although only two out of the three criteria are needed to make a diagnosis, yellow nail discoloration and dystrophy should be one of them [4]. There is no precise data available about its prevalence, but it is estimated to be <1/1,000,000 without specific geographical distribution or a population at higher risk. Its etiology is still not well defined; however, lymphatic drainage dysfunction versus microvascular disease associated with increased permeability and protein leakage are the main two theories that may explain its pathophysiology. [3,5]

The most remarkable and unique feature of this syndrome is the yellow nail discoloration and dystrophy. The nail plate becomes slow-growing, more curved, harder to be trimmed, and plate-bed separation occurs. It may resolve spontaneously especially in fingernails rather than toenails, which may be explained by lymphatic dysfunction and lower limbs lymphedema. [6,7]

Chronic cough is the most common respiratory presentation in this syndrome as it is reported in more than half of cases. Unilateral pleural effusion is reported in around 40% of cases and bilateral in 60% of cases, serous in 75%, chylous in 22%, and exudative in 95% of cases, with an average protein level of around 4 g/dl. Pleural biopsies are not conclusive, and they either show a normal histological picture or a picture of chronic inflammation [1].

Bronchiectasis is reported in 44% of cases. Although the same bacterial spectrum of idiopathic bronchiectasis (*Pseudomonas aeruginosa*, *Haemophilus influenzae*, *Streptococcus pneumoniae*, *Moraxella catarrhalis*) was isolated from sputum, the bronchiectasis in yellow nail syndrome is reported to be less severe than that of idiopathic cases [8].

Bilateral lower limb lymphedema is reported in 30-80% of cases. Accumulation of lymph in subcutaneous tissue results in stimulation of fibroblasts and adipocytes leading to fibrosis and more fat deposition. This is usually complicated with cellulitis and poor quality of life [9-11].

Although acute or chronic rhinosinusitis is not included in the criteria of yellow nail syndrome diagnosis, it is commonly reported in association with it. It usually presents with nasal obstruction, mucopurulent nasal discharge, and postnasal drip.

Yellow nail syndrome has no available curative treatment and some of the cases resolve spontaneously with symptomatic treatment within few months.

With regard to nail discoloration and dystrophy, oral vitamin E is reported to be effective in treatment, most likely due to its antioxidant properties that may prevent the formation of lipofuscin pigments, which are thought to be the cause of discoloration. Azole antifungals are sometimes prescribed in nails treatment, not because of their antimicrobial properties, but for their stimulatory action on linear nail growth. However, it shows partial efficacy and it was not used in this case [12,13].

Regarding pulmonary manifestations, VATS followed by decortication or pleurodesis is the intervention of choice for recurrent and/or large pleural effusions. Octreotide was used in some cases and showed promising responses. Management of patients presenting with bronchiectasis is the same as that of idiopathic, including antibiotics targeting the highly suspected organisms, chest physiotherapy, and postural drainage [8,14].

Lymphedema is treated by a multimodal decongestive therapy which is achieved in two phases. Low stretch bandages, manual lymph drainage, and physical exercises are used to achieve lymphedema volume reduction - the target of the first phase. This is followed by the second phase which aims at long-term stabilization of lymphedema volume by wearing high elastic stocking, exercise, and skincare. This improves the quality of life and avoids further complications, e.g., cellulitis [15,16].

Unfortunately, treatment of the associated rhinosinusitis with usually used medications, i.e., antibiotics, intranasal steroids, and decongestants, has shown poor response, and that is why surgical procedures are sometimes needed [17].

## Conclusions

This is a case of yellow nail syndrome, an extremely rare condition of unknown etiology, presenting with a clinical triad of yellow discoloration and dystrophy of nails, lymphedema, and recurrent pulmonary manifestations. Lymphatic drainage dysfunction versus micro-vasculopathy with protein leakage may explain the syndrome's pathogenesis. No available definitive treatment is available and the treatment centers on controlling symptoms. Physicians need to be aware and mindful of the syndrome to make a diagnosis. More research is needed for a better understanding of its pathophysiology and to reach a definitive curative treatment.
